# Somatic extracts of *Marshallagia marshalli* downregulate the Th2 associated immune responses in ovalbumin-induced airway inflammation in BALB/c mice

**DOI:** 10.1186/s13071-017-2159-8

**Published:** 2017-05-12

**Authors:** Sima Parande Shirvan, Azadeh Ebrahimby, Arezoo Dousty, Mohsen Maleki, Ahmadreza Movassaghi, Hassan Borji, Alireza Haghparast

**Affiliations:** 1 Department of Pathobiology, Faculty of Veterinary Medicine, Ferdowsi University of Mashhad, P. O. Box: 91775-1793, Mashhad, Iran; 2 Division of Biotechnology, Faculty of Veterinary Medicine, Ferdowsi University of Mashhad, P. O. Box: 91775-1793, Mashhad, Iran

**Keywords:** Allergic asthma, Helminth therapy, Immunomodulation, *Marshallagia marshalli*, Somatic products

## Abstract

**Background:**

Recently the role of gastrointestinal nematodes in modulating the immune responses in inflammatory and immune-mediated conditions such as allergy and autoimmune diseases has been introduced. This is mainly due to the suppressive effects of somatic and excretory secretory (ES) products of nematodes on the immune responses. In this study, we evaluated the immunomodulatory potentials of somatic products of *Marshallagia marshalli*, a gastrointestinal nematodes of sheep, to suppress the immune-mediated responses in a murine model of allergic airway inflammation. BALB/c mice were intraperitoneally (IP) sensitized with ovalbumin (OVA)/Alum and then challenged with 1% OVA. Somatic products of *M. marshalli* were administered during each sensitization. The effects of somatic products on development of allergic airway inflammation were evaluated by analyzing inflammatory cells recruitment, histopathological changes, cytokines production (IL-4, IL-13, IL-10, TGF-β) and serum antibody titers (IgG1, IgG2a).

**Results:**

Somatic products of *M. marshalli* were able to suppress the induction of allergic airway inflammation in mice. Modulation of Th2 type responses (IL-4, IL-13, IgG1) *via* upregulations of IL-10 and TGF-β production was observed after injection of somatic products of *M. marshalli*. In addition, inflammatory cells infiltration and pathological disorders were significantly diminished following administration of somatic products.

**Conclusions:**

Our data raised the possibility that helminths could be a potential therapeutic candidate to alleviate the inflammatory conditions in allergic asthma. According to these results, we concluded that *M. marshalli* may contain immune-modulatory molecules that attenuate allergic airway inflammation *via* induction of regulatory cytokines. Further investigations are required to identify molecules that might have potentials for development of novel therapeutic targets.

## Background

Allergic asthma is an inflammatory disease that affects millions of people in industrial countries. However, the prevalence of this disease has been increased in developing countries over the last few decades [1, 2]. Although there are differences in the global prevalence, the burden of allergic asthma is on rise [3]. While allergic asthma emerges as global threats, and with awareness of the limitation of available treatments and some cases of corticosteroids resistance asthma [4], there is an essential need to develop advanced and novel treatments. The most effective way to control asthma is to remove the source of allergen. However, allergen avoidance is not always protective against sensitization and development of the disease [5] and it is virtually impossible that people with asthma avoid all triggers of this condition. Although asthma is a heterogeneous disorder, a dysregulated Th2 immune response to allergens is present in most subsets of asthma [2]. Recent evidence has revealed the contribution of other T cell subsets such as Th17, Th9 and CD8^+^ T cells in the development of allergic asthma [6]. Development of novel, targeted therapeutics such as humanized monoclonal antibodies (Mepalizumab [7] and Tocilizumab [8]) have been reported in the control of severe eosinophillic asthma. However, these products have not been approved for use in any other eosinophil-related disorders. Induction of regulatory responses is highly desirable for development of therapeutic targets against allergic airway inflammation [9]. Omalizumab is a recombinant humanized monoclonal antibody that has shown a positive effect on food allergy [10], severe allergic asthma [11] and overlapping COPD [12]. Although Omalizumab reduces circulating IgE and provoke IL-10 production [13], it has been used as a treatment to reduce inhaled corticosteroid and still adverse effects are reported [14]. Therefore, new studies are needed to find products to cure or prevent asthma.

It is established from a wealth of literature that helminths, in addition to Th2 responses, can induce regulatory T (Treg) cell responses, which contribute to ensure their survival in the host [15]. In fact, during early exposure to allergen, helminth-derived products train regulatory T cells, leading to inappropriate immune stimulation. This phenomenon induces immune tolerance that establishes a balance between inflammatory and anti-inflammatory responses [16]. Regulatory responses during early exposure to allergens are important in immune priming and could orchestrate airway inflammation, leading to reduce the incidence of allergic asthma [17, 18]. Therefore, helminths may be an attractive therapeutic option, if they reduce airway inflammation.

Both epidemiological [19] and experimental [20, 21] studies have revealed that exposure to helminth infection evoke suppressive effects on the development of a broad spectrum of immune-mediated diseases such as allergy and autoimmune diseases. These results provide a supportive evidence for confirmation of hygiene hypothesis [22], the proposition that helminth infection can impair immunological reactivity. In addition, these results provide new insights into using helminth products in treatment of immune-mediated diseases.

Recently, helminth therapy has been successfully used in animal models of allergic asthma [23] and autoimmune diseases such as inflammatory bowel disease (IBD) [24, 25], experimental autoimmune encephalomyelitis (EAE) [26] and type 1 diabetes [27]. In addition, helminth have been prescribed in controlled clinical trials of multiple sclerosis (MS) [28, 29] and IBD [30, 31]. However, the use of helminths for the treatment of inflammatory diseases has several drawbacks, such as iatrogenic infection with non-adaptive worms in human, anaphylactic reactions [32], prolonged chronic infection, and side effects of natural worm infections [33]. Susceptibility to viral, bacterial and protozoan infections in regards to general suppression of immune responses is also another concern associated with helminth therapy [17]. Other limitations of helminth therapy may include the high cost of germ-free eggs or larval preparation. In addition, patients may be reluctant to consume eggs and larva. Therefore, one way to possible enlarge the market of helminths and solve the adverse problems with live worms would be the use of helminth-derived products that have anti-inflammatory potential [34, 35]. Although the use of helminth-derived products is yet to be tested, these products seem to have less controversial and more patient compliance. Given the previous studies showing anti-inflammatory properties of helminth-derived products [36], the use of these products enhance the potential for future therapies to prevent the development of allergic asthma.

*Marshallagia marshalli* is a nematode parasite from the family Trichostrongylidae that inhabits in sheep abomasum. This nematode is worldwide distributed and is important in tropical and subtropical climates. In northeast of Iran where this study was conducted, *M. marshalli* is one of the most important and prevalent helminths that infect ruminants [37]. Short lifespan, easy accessibility and our recent study on this nematode [38] alongside with the growing evidence of using helminths as potential immunomodulators in reducing the airway inflammation in animal models, made us to conduct this study to assess the immunoprophylactive effects of *M. marshalli* somatic products on attenuating the Th2-driven allergic airway inflammation in a murine model of ovalbumin (OVA) induced allergic asthma.

## Methods

### Animals

Six to eight week-old female BALB/c mice were purchased from Razi Vaccine and Serum Research Institute, Mashhad, Iran, and maintained in animal housing facilities at Ferdowsi University of Mashhad throughout the experiment. Free access to water and food was provided to animals during the experiments.

### Study design

To evaluate whether somatic extract of *M. marshalli* modulate the onset of allergic airway inflammation, two independent experiments was designed. In each experiment, mice were randomly divided into three groups (5 mice per group). The first group sensitized and challenged with phosphate buffer saline (PBS). The second group (OVA-sensitized mice): sensitized with OVA + Alum and challenged with OVA. The third group (somatic administered group): mice that received 20 μg somatic extract of *M. marshalli* during sensitization along with OVA + Alum and challenged with OVA.

### Preparation of *M. marshalli* somatic extract

At the post-mortem, helminths were harvested from sheep abomasum. Adult worms were isolated and identified based on the morphological features under a light microscope. Collected worms were washed several times and homogenized with sterile PBS containing antibiotics (penicillin/streptomycin) to a stable homogenous state while on ice. The worm homogenate was centrifuged at 10,000× *g*, 4 °C for 10 min and the supernatants were collected into new sterile tubes. Afterwards the supernatants were passed through 0.2 μm filter and the total protein concentration was determined by Bradford assay (Bio-Rad, Hercules, CA, USA). Prepared somatic extract was aliquated and stored at -80 °C until use.

### Murine model of OVA induced airway inflammation

**Fig. 1:**
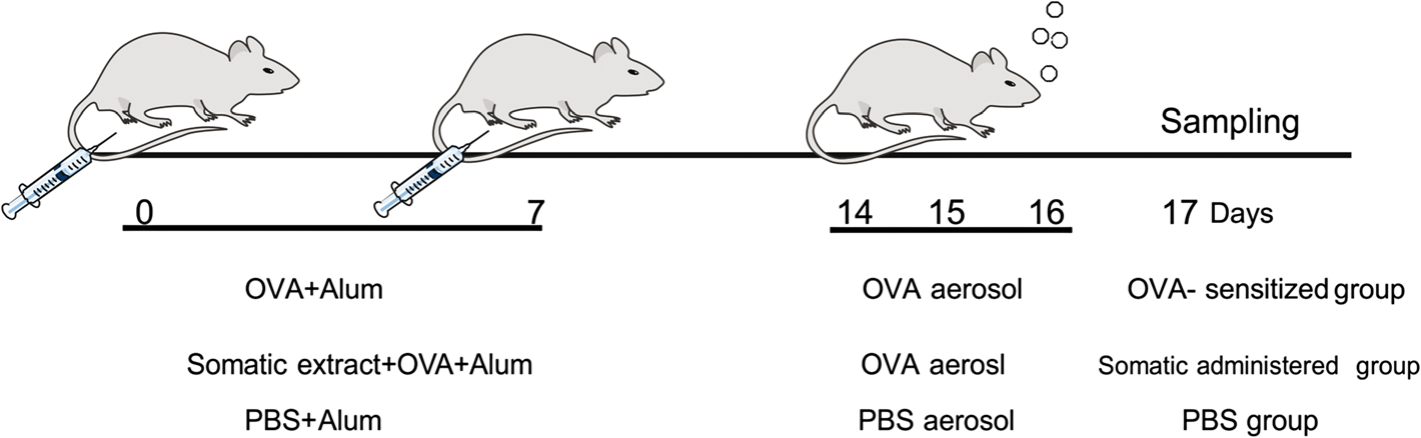
Experimental protocol for induction of allergic airway inflammation and treatment scheme

Induction of allergic airway inflammation was performed as previously described by Smits et al. [39]. Briefly mice were sensitized intraperitoneally (IP) with two injection of OVA (10 mg/ml, Worthington Biochemical Corp, NJ, USA) emulsified in AL(OH)3 (1 mg; Imject Alum, Pierce, Rockford, IL, USA) on day 0 and day 7. The somatic administrated group received 20 μg somatic extract during sensitization with OVA-Alum. The PBS group was sensitized with 0.5 ml PBS-alum in the same days on days 0 and 7. Mice were then challenged with aerosol administration of 1% OVA at days 14, 15 and 16. For this purpose, mice in OVA sensitized and somatic administrated groups were transferred into aerosol boxes and challenged with 1% OVA protein in PBS for 30 min. This procedure was repeated three times in three consecutive days. PBS group were challenged by nebulizer inhalation of PBS at the same times. Twenty-four h after the last challenge mice were sacrificed after anaesthetizing with ketamin and xylazin (Fig. 1).

### Collection of bronchoalveolar lavage (BAL) fluid for total cell counts

To collect BAL fluid the tracheas were cannulated and 3 × 0.4 ml PBS was instilled into lungs. The lavage fluid was withdrawn by negative pressure. A total of 1 ml BAL fluid was collected and centrifuged at 400 *g* at 4 °C for 10 min. The cell free supernatants were stored at -20 °C for subsequent analysis and the cell pellets were resuspended in 100 μl PBS; total inflammatory cells in the BAL fluid were enumerated using a hemocytometer.

### Preparation of lung homogenate

After BAL fluid collection, the lungs were harvested and divided in two sections. One part was fixed in 10% phosphate-buffered formalin for histopathological analysis. Second part of lungs was frozen in liquid nitrogen and stored at -80 °C until cytokine detection was performed. On the day of analysis, frozen lungs were thawed and weighed. 100 mg of wet tissue was homogenized with a tissue homogenizer in 1 ml homogenization buffer containing KCL (0.5 M), Tris-CL (1 M, pH 9) and Triton X-100. The homogenates were centrifuged at 10,000× *g* for 10 min at 4 °C. The supernatants were used for cytokine detection by enzyme linked immunosorbent assay (ELISA).

### Histopathological analysis of lung tissue

After 24 h of formalin fixation, lung tissues were embedded in paraffin, cut into 3–5 μm sections and stained with hematoxylen eosin (H & E) to assess the degree of inflammatory cells infiltration. The tissue sections were also stained with periodic acid-Schiff stain (PAS) to observe the presence and intensity of goblet cells and mucus.

For quantification of pathological disorders a grade pattern was used to illustrate a histologic score, ranging from 0 to 3 as previously described [40]. Increasing grade was directly related to asthma severity. The pathology slides were blindly assessed by two expert pathologists for the grading of asthma severity based on the intensity of inflammatory cells infiltration as well as goblet cells metaplasia. The following grading was applied: Grade 0 (absent): lack of infiltration of inflammatory cells and normal distributions of goblet cells in lung tissue; Grade 1 (mild): slight infiltration of inflammatory cells and goblet cells metaplasia; Grade 2 (moderate): moderate infiltration of inflammatory cells and goblet cells metaplasia; and Grade 3 (severe): strong inflammatory cells infiltrations and goblet cells metaplasia.

### Cytokines detection

Levels of IL-4, IL-13, TGF-β and IL-10 were detected in BAL fluid and lung tissue homogenates by sandwich ELISA using DuoSet kits as recommended by the manufacturer (R & D Systems Inc., Minneapolis, MN, USA) instruction. In brief, 96-well plates were coated with capture Ab (R & D Systems Inc., Minneapolis, MN, USA), overnight at 4 °C. Plates were blocked with bovine serum albumin (BSA) for 2 h at room temperature. Afterwards, 100 μl sample was added to plates and incubated at room temperature for 2 h. Detection was performed with detection Ab (R & D Systems Inc., Minneapolis, MN, USA), followed by addition of streptavidin-HRP and substrate. The reaction was stopped by adding the stop solution and ODs was read at 450 nm in an ELISA plate reader (ELX800 absorbance reader, Bio TeK, USA). Detection limits were 15.60 pg/ml, 62.50 pg/ml for IL-4 and IL-13, respectively and 31.20 pg/ml for IL-10 and TGF-β.

### Detection of IgG antibody isotypes

On the day of sacrifice, blood samples were taken and sera were stored in -20 °C. IgG1 and IgG2a concentration were detected in serum by sandwich ELISA according to the manufactures instruction (eBioscience, Inc., San Diego, CA, USA). Detection limits for IgG1 and IgG2a was 3.13–200 ng/ml and 3.90–250 ng/ml, respectively.

### Statistical analysis

All data were analyzed using Prism 6.01 (Graghpad, La Jolla, CA, USA) software. Statistical significance of differences between groups was determined using one-way ANOVA and Kruskal-Wallis tests. *P*-values < 0.05 were considered significant.

## Results

### Somatic extract of *M. marshalli* suppressed the development of OVA-induced allergic airway inflammation

Based on our knowledge there is no experimental evidence showing the therapeutic effects of *M. marshalli* somatic products in acute allergic airway inflammation. Therefore, we examined the possible suppressive capacity of somatic extract derived from this nematode on development of acute airway inflammation in mice.

**Fig. 2:**
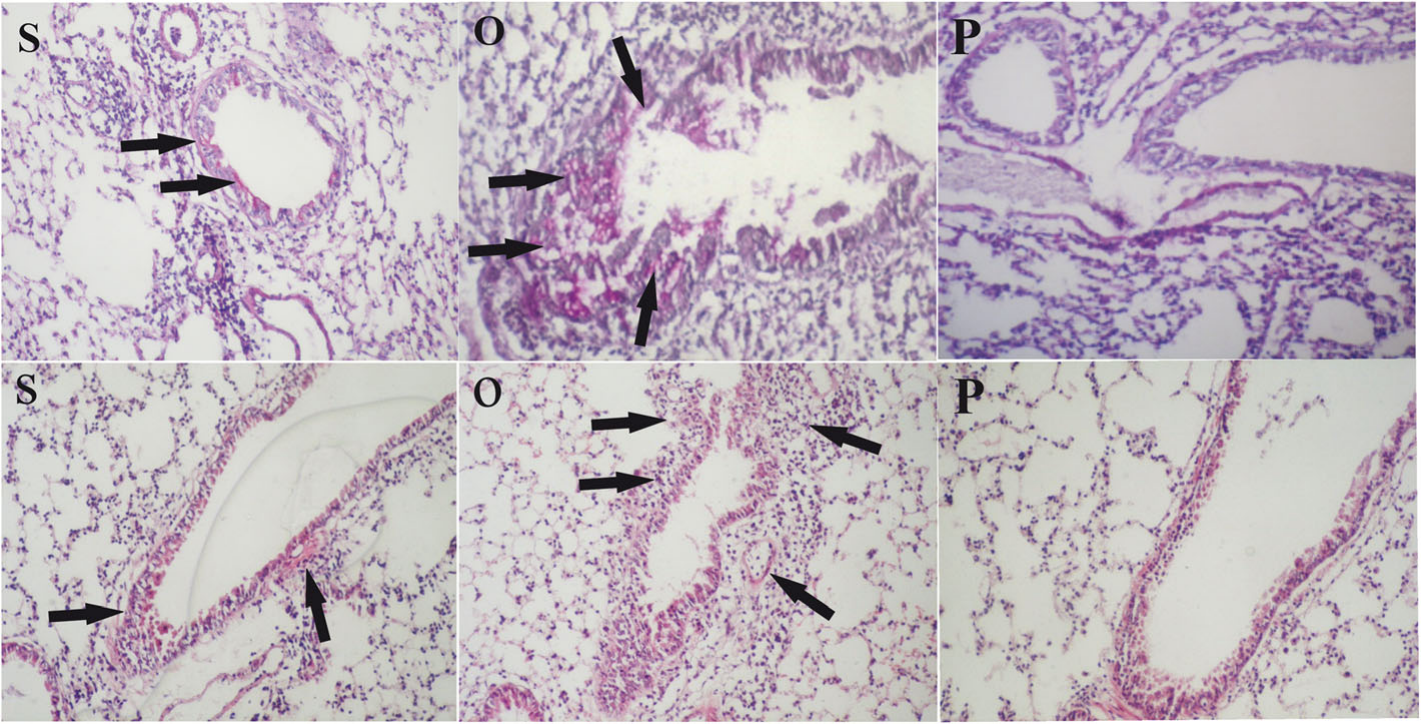
Somatic extract of *M. marshalli* abrogates the development of allergic airway inflammation. Mice received OVA/alum or PBS on days 0 and 7 and challenged using nebulization with 1% OVA or PBS for 30 min, three times over the course of experiment. In addition, some mice were treated with 20 μg of somatic extract during sensitization with OVA and then challenged with 1% OVA. Mice were sacrified at day 17, and lung sections were prepared and stained with periodic acid Schiff staining (PAS) and hematoxylin and eosin (H&E) for observation of goblet cells metaplasia and inflammatory cells infiltration, respectively. *Abbreviations*: O, OVA-administered mice group (*arrows * indicate severe infiltration of inflammatory cells goblet cells metaplasia); S, somatic + OVA administered mice (arrows indicate mild infiltration of inflammatory cells and goblet cells metaplasia); P, PBS received mice (no inflammatory cells infiltration and goblet cells metaplasia)

**Fig. 3:**
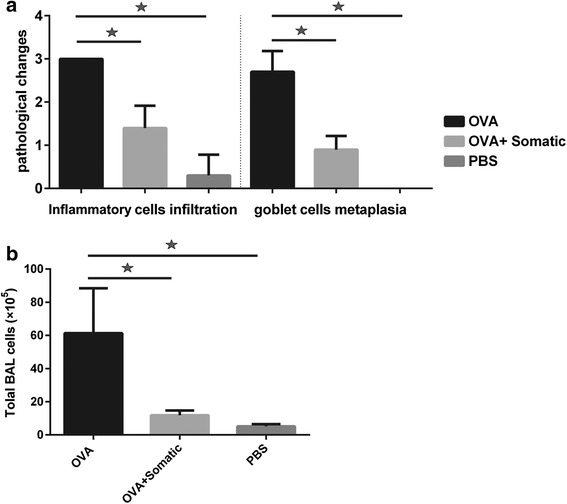
Therapeutic potentials of *M. marshalli* on development of OVA-induced allergic asthma. 20 μg of somatic extract of *M. marshalli* was administrated during each sensitization with OVA and then aerosol challenges were performed using 1% OVA. Bronchioalveolar lavage (BAL) and lung tissue were collected at day 17. **a** Frequency of histopathological scoring (H & E and PAS) of asthma, statistical analysis was performed with the Kruskal*-*Wallis test. Data represent mean and SEM of five mice, with a mean score from three lung section per mouse. *P*-value < 0.05. **b** Inflammatory cells count in the BAL fluid. Error bars show SD. *P* < 0.05; one-way ANOVA test

After 24 h of allergen (OVA) challenge, OVA-sensitized mice developed allergic airway inflammation as characterized by intense infiltration of inflammatory cells, predominantly eosinophils, into lung. In contrast, no inflammatory cells infiltration was observed in the control group (sensitized and challenged with PBS). In the treatment group, two administrations of somatic products of *M. marshalli* during sensitization with OVA significantly decreased inflammatory cells infiltration (Kruskal-Wallis: *χ* ^2^ = 25.7, *df* = 2, *P* < 0.0001) (Figs. 2, 3a). PAS staining was performed to evaluate the effects of somatic extract of *M. marshalli* on the goblet cells metaplasia and hyperplasia in bronchioles and bronchi, respectively. OVA-induced mice as compared with PBS group developed severe goblet cells metaplasia and hyperplasia, as well as mucus secretion in bronchi. The presence of somatic products during OVA sensitization substantially decreased PAS-positive goblet cells, resulting in reduced mucus production (Kruskal-Wallis: *χ* ^2^ = 26.982, *df* = 2, *P* < 0.0001) (Figs. 2, 3a).

### The numbers of total inflammatory cells and eosinophils were decreased in BAL fluid of mice received somatic products plus OVA during each sensitization

We performed cell counting to investigate the presence and intensity of inflammatory cells into BAL fluid in the experimental groups. Total cell counts in BAL fluid showed the same pattern as pathological disorders in the experimental groups. Mice received OVA displayed the highest total number of cells in BAL fluid, which was due to the increasing number of eosinophils. In contrast, as shown in Fig. 3b, somatic extract greatly affected the allergic response by decreasing inflammatory cells infiltration into BAL fluid (ANOVA: *F*_(2, 24)_ = 34.10, *P* < 0.0001).

### Administration of somatic extract of *M. marshalli* suppressed Th2 cytokines production

**Fig. 4:**
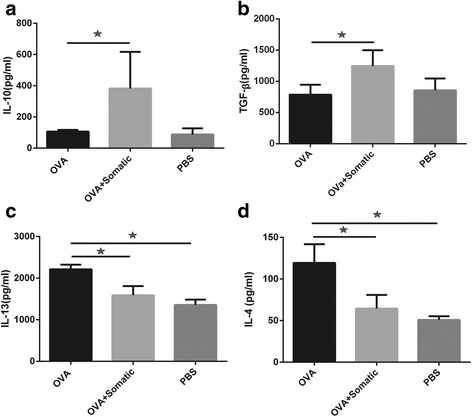
Somatic extract of *M. marshalli* significantly suppressed Th2 cytokines *via* upregulation of IL-10 and TGF-β. Mice received somatic extract of *M. marshalli* during each sensitization and challenged with OVA on days 14, 15 and 16. Lung tissues were collected at day 17 and homogenized on the day of analysis. Cytokine responses (**a** IL-4, **b** IL-13, **c** IL-10, **d** TGF-β) were measured in mice given OVA/Alum in the presence or absence of somatic products. Error bars are SD. *P* < 0.05; one-way ANOVA

To elucidate the suppressive effects of somatic extract on development of immune responses, the Th2 cytokine levels were measured by ELISA. IL-4 and IL-13 levels were significantly increased in OVA sensitized mice, as compared to the PBS group (ANOVA: *F*_(2, 24)_ = 44.69, *P* < 0.0001 and ANOVA: *F*_(2, 27)_ = 75.51, *P* < 0.0001, respectively). In line with less inflammatory cell infiltration into the lung, the IL-4 and IL-13 levels in lung homogenate supernatants were decreased following somatic extract administration (Fig. 4a, b). Surprisingly, no significant differences were seen between groups in terms of cytokine levels in BAL fluid. Furthermore, IL-4 was not detectable in BAL fluid (data not shown).

### Treatment of mice with somatic extract conferred protection from allergic asthma by inducing immunoregulatory cytokines

To address whether regulatory responses are evoked with somatic administration, we measured IL-10 and TGF-β levels in lung homogenates. As expected, the suppressive effects of *M. marshalli* somatic extract on OVA-induced airway inflammation were significantly associated with upregulation of IL-10 (ANOVA: *F*_(2, 27)_ = 14.46, *P* < 0.0001) and TGF-β (ANOVA: *F*_(2, 24)_ = 13.27, *P* = 0.0001) levels (Fig. 4c, d).

### Somatic extract of *M. marshalli* suppressed IgG1 isotype level

**Fig. 5:**
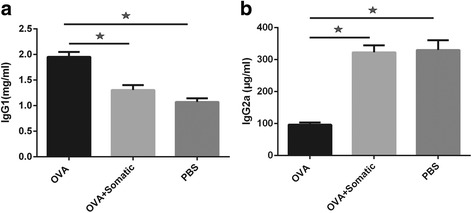
Somatic extract of *M. marshalli* significantly suppressed IgG1 isotype level. Total IgG1 (**a**) and IgG2a (**b**) were measured in the serum to identify the effects of somatic extract of *Marshallagia marshalli*. The mean values were shown for five mice per group. Error bars are SD. *P* < 0.05; one-way ANOVA

Similar to Th2 associated cytokines results, immunization with OVA led to higher levels of IgG1 antibody production in comparison to PBS (ANOVA: *F*_(2, 21)_ = 207.2, *P* < 0.0001). The administration of somatic extract significantly decreased the induction of IgG1 antibodies in sera of mice during sensitization with OVA (Fig. 5a). IgG2a levels were lower in mice received OVA, as expected (ANOVA: *F*_(2, 21)_ = 290.9, *P* < 0.0001). However, no significant differences were observed between mice received somatic extracts and PBS (Fig. 5b).

## Discussion

During the past few years, helminth administration in a murine model of immune-mediated disease has been considered substantially, providing new treatment approaches in allergic asthma and other immune-mediated diseases. In this regard, experimental studies have been initiated to evaluate efficacy and safety of helminth therapy in a murine model of allergic asthma [41], IBD [24], MS [26] and type 1 diabetes [27]. The findings are consistent with epidemiological studies showing a negative relationship between helminth infection and allergic diseases [19]. In addition, clinical trials elucidated the therapeutic impacts of *Trichuris suis* ova on IBD [30, 31, 42] and MS patients [28, 29]. Anti-inflammatory protein-2 (AIP-2), a hookworm secreted protein, suppressed airway inflammation in a mouse model of allergic asthma, decreased expression of co-stimulatory molecules on human DCs and suppressed proliferation of *ex vivo* peripheral blood mononuclear cells (PBMCs) from human subjects with house dust mite allergy [43]. However, the role of helminths in treatment of asthma is still contradictory. Some population surveys have shown the protective effects of helminth infection and increased positive skin prick following antihelminthic therapy [44], while other studies have found a positive association between helminth infection and asthma severity [45].

Although, many studies have shown the anti-inflammatory potential of murine helminths in a murine model of airway inflammation, a few reports have been published with regard to the administration of non-murine helminths in a murine model of airway inflammation [21, 41].

To our knowledge, our study revealed for the first time the prophylactic potentials of somatic extract of a ruminant nematode, *M. marshalli*, in a murine model of allergic airway inflammation. In particular, administration of somatic extract of *M. marshalli* during OVA sensitization resulted in reduction of Th2 associated immune responses including IL-4 and IL-13 and IgG1, as well as inflammatory cells infiltration, eosinophilia and goblet cells hyperplasia. These findings are consistent with previous animal studies [23, 46, 47].

Th2 associated cytokines including IL-4 and IL-13 are upregulated in both helminth infections and allergic disorders [48, 49]. These cytokines are involved in the pathophysiology of asthma. IL-4 facilitates T cell priming towards new antigens [50] and mediates isotype switching in B cells [51]. IL-13 is associated with mucus production and goblet cells hyperplasia in airway epithelium cells [52] and is a key driver of airway inflammation [53]. Studies predict significant clinical effects with anti-cytokine therapies that abrogate the production of Th2 associated cytokines [54, 55] such as Lebrikizumab [56]. Therefore, we hypothesized that somatic extract of *M. marshalli* might decrease IL-4 and IL-13 levels when administrated during OVA sensitization. Our data showed the suppressive capacity of somatic administration on IL-4 and IL-13 levels in lung homogenate supernatants. In accordance with IL-13 levels, somatic administered group revealed fewer PAS-positive goblet cells. However, no significant differences were seen between groups in terms of cytokine levels in BAL fluid. Furthermore, IL-4 was not detectable in BAL fluid (data not shown). Given that IL-4 is localized in airway epithelium with respect to high affinity IL-4 receptors on airway epithelium, it is supposed that the time of mice scarification is a crucial factor in cytokines detection in BAL fluid. McSorley et al. [23, 57] showed the time-dependent of cytokine changes in BAL fluid and peritoneal lavage supernatants, revealing a decrease of cytokine levels over a time course post-administration. Therefore, we are not able to make any conclusion about non-significant differences between groups in cytokine levels of BAL fluid.

A number of immune responses associated with helminths have been proposed to prevent excess inflammation and allergic diseases. Because asthma is associated with aberrant Th2 responses, induction of regulatory responses or Th1 responses can suppress the development of allergic airway inflammation. Kim et al. [41] showed a shift from Th2 to Th1 response in mice sensitized with OVA and somatic extract of *Caenorhabditis elegans*. Moreover, administration of IFN-γ into the airway prior to and during primary OVA sensitization inhibited the development of OVA-induced airway inflammation. Treatment with IFN-γ also prevented the development of anti-OVA responses after establishment of primary allergic reactions [58]. In the present study the level of IgG2a which is associated with Th1 responses was lower in OVA-sensitized mice which was in accordance with high levels of IgG1 and Th2 immune responses. However, somatic and PBS administered groups exhibited increased level of total IgG2a production in sera, indicating a marked shift towards Th1 responses (Fig. 5b). Although there was a slight decrease in IgG2a production in somatic administered group as compared to PBS group, this finding was not significant (Fig. 5b). Given the non-significant difference between PBS and somatic administered group in IgG2a production, a Th1 biased response due to somatic products could not concluded from this finding.

Another possible mechanism in suppression of allergic asthma by somatic extract of *M. marshalli* might be related to immunregulatory responses. Immunoregulatory cytokines have been implicated in parasite-derived modulation of host immunity during chronic infection [15, 36]. It was suggested that parasite-derived regulatory cytokines may be involved in blocking inflammatory responses such as allergic asthma [17, 20]. This hypothesis was supported by demonstrating that administration of somatic products generated significantly higher levels of IL-10 and TGF-β (Fig. 4 c, d). In accordance with our results, previous studies showed the therapeutic potentials of helminths in resistance from allergic asthma *via* the expansion of CD4^+^CD25^+^FOXP3^+^ regulatory cells and IL-10 and TGF-β production [21, 59]. For example, the larval ES products of *T. suis* have immunomodulatory functions and could prevent OVA-induced allergic asthma in a IL-10 dependent manner [20]. Filarial cystatin, a secreted protease inhibitor of filarial nematodes (*Acanthocheilonema viteae*) reduced IL-4 production and suppressed OVA-induced allergic airway inflammation through a mechanism involving anti-inflammatory cytokine IL-10. Blocking of IL-10 restored the suppressive effects of filarial cystatin while depletion of regulatory cells had a limited effect [60]. S*chistosoma japonicum* HSP60-derived peptide modulated the effector responses against OVA induced delayed-type hypersensitivity through a mechanism involving anti-inflammatory cytokines IL-10 and TGF-β [61]. However, the secreted products of *Heligmosoides polygyrus* could protect mice from allergic asthma *via* Treg independent mechanisms. This suppression was attributed to reduced group 2 innate lymphoid cells and type 2 inflammation in the lung [23, 62]. In particular, regulatory cells induced by helminths inhibit recruitment and activation of effector cells [63]. Furthermore, IL-10 and TGF-β secreted by regulatory T cells skew the antibody production from IgE towards IgG4 and IgA [64]. There are several sources of IL-10 and TGF-β production including regulatory T cells [65], regulatory B cells [66] and alternative activated macrophages (AAM) [15]. Although our results implicated that somatic extract of *M. marshalli* could induce immunoregulatory cytokines, resulting in suppression of asthma progression, further investigations should be performed to find the mechanisms of protection from allergic inflammation by *M. marshalli*.

## Conclusions

Our results add credence to the current investigation of using helminths in asthma treatment. We showed the beneficial effects of *M. marshalli* somatic extracts in attenuation of OVA-induced allergic airway inflammation *via* upregulation of IL-10 and TGF-β production. This suppression involved both antibody production (IgG1) and cellular responses (eosinophils infiltration and goblet cell metaplasia). These findings provide new insights into development of biological therapy in allergic asthma and that this nematode would appear of particular interest as potential treatment of acute airway inflammation.

## Abbreviations

AAM: Alternative activated macrophages
EAE: Experimental autoimmune encephalomyelitis
ES: Excretory-secretory
IBD: Inflammatory bowel disease
MS: Multiple sclerosis
OVA: Ovalbumin
Treg: Regulatory T cell
